# Micropatterning of 3D Microenvironments for Living Biosensor Applications

**DOI:** 10.3390/bios4010028

**Published:** 2014-02-27

**Authors:** William F. Hynes, Nate J. Doty, Thomas I. Zarembinski, Michael P. Schwartz, Michael W. Toepke, William L. Murphy, Sarah K. Atzet, Ryan Clark, J. Andres Melendez, Nathaniel C. Cady

**Affiliations:** 1 State University of New York (SUNY) College of Nanoscale Science & Engineering, 237 Fuller Road, Albany, NY 12203, USA; E-Mails: rhynes@albany.edu (W.F.H.); rclark@albany.edu (R.C.); jmelendez@albany.edu (J.A.M.); 2 BioTime, Inc., 1301 Harbor Bay Parkway, Alameda, CA 94502, USA; E-Mails: ndoty@biotimemail.com (N.J.D.); tzarembinski@biotimemail.com (T.I.Z.); skfarney@gmail.com (S.K.A.); 3 Wisconsin Institutes of Medical Research, University of Wisconsin-Madison, 1111 Highland Ave, Madison, WI 53705, USA; E-Mails: mpschwartz@wisc.edu (M.P.S.); mike_toepke@hotmail.com (M.W.T.); wlmurphy@wisc.edu (W.L.M.)

**Keywords:** microprinting, biosensor, hydrogel, reactive oxygen species, roGFP-R12, ROS, hyaluronan, hyaluronic acid, gelatin, PEG norbornene, Irgacure

## Abstract

Micro-scale printing and patterning of living cells has multiple applications including tissue engineering, cell signaling assays, and the fabrication of cell-based biosensors. In this work, a molecular printing instrument, the Bioforce Nano eNabler, was modified to enable micron-scale “quill-pen” based printing of mammalian cells in a 3D hyaluronan/gelatin based hydrogel. Specifically, photo-initiated “thiol-ene” click chemistry was used to couple the thiol groups of thiolated hyaluronan/thiolated gelatin to the alkene groups of 4-arm polyethylene glycol (PEG)-norbornene molecules. Rapid photopolymerization enabled direct printing and controlled curing of living cells within the hydrogel matrix. The resulting hydrogels were biocompatible with human adipose-derived stem cells, NIH-3T3 cells, and mouse embryonic stem cells. The utility of this printing approach was also explored for cell-based biosensors. Micro-printed cells expressing a redox sensitive variant of the green fluorescent protein (roGFP-R12) showed a measurable fluorescent response to addition of oxidizing and then reducing agents. This work represents a novel approach to micron-scale cell patterning, and its potential for living, cell-based biosensors.

## 1. Introduction

Bioprinting is an expanding field that addresses the growing requirement for the precise spatial arrangement of live cells and biomaterials onto solid and semi-solid surfaces [1]. Currently, most of the existing bioprinting techniques are focused on tissue engineering applications, where the techniques are used to deposit cells and scaffolding materials in specific patterns [2]. This can, in turn, be used to engineer organ- and tissue-like constructs, for the purpose of *in vitro* drug testing and disease progression modeling [3]. There are other less explored applications for bioprinting which require the precise positioning of cells and materials, including high-throughput drug screening platforms and fabrication of live-cell-based biosensors. By utilizing bioprinting to deposit microarrays of cells, drugs, or other analytes, rather than using bulk cell cultures, these screening and sensing platforms can be effectively miniaturized to the point where the necessary volumes of these materials are substantially less than what would be required to elicit a response from a bulk culture [4]. In addition to saving on material costs for reagents, patterning cells into arrays would allow for simple observation and tracking of individual cells throughout the trials, rather than attempting to deconvolute an individual cell’s response from a larger population. Standard bulk cultures allow little to no spatial control of cell or signaling factor distribution, which can cause a situation where local signal strength and individual cell response cannot be readily correlated. In order to design an effective platform for the study of cell signaling phenomena, variables such as cell position, cell density, signal concentration and signal gradient formation must be well controlled. Spatial control over cell and biomolecule deposition makes bioprinting attractive to tissue engineers [5]. Signal gradients can be established by patterning cells and bio-active molecules in specific geometries upon a planar surface, resulting in a simplified, yet versatile platform to monitor the activity of the exposed cells. Real time monitoring of cellular physiology using bioprinting could also elucidate the mechanism of action of the drug or signal within the cells, if the proper measures are taken to monitor the resulting intracellular events upon exposure.

One unique application for bioprinting is the fabrication of cell-based biosensors. Cell-based biosensors have exceptional promise for applications in cytotoxicity screening due to their ability to sense the presence of toxins or stressors in the environment, while also demonstrating the bioavailability and cytotoxic effects of the toxins present [6,7,8,9,10]. Cell-based biosensors have the potential to give researchers quantitative data on the concentrations of available toxicant within the cellular microenvironment [11]. In this work we have targeted monitoring of concentration shifts for reactive oxygen species (ROS), which can result from changes in the steady state levels of hydrogen peroxide (H_2_O_2_). ROS are normal byproducts of healthy cell metabolism and have important roles in intracellular signaling pathways such as the activation of NFκB/rel family transcription factors [12] and regulating the cell’s redox equilibrium [13]. Currently, fluorescent, redox-sensitive intracellular probes, such as roGFP, are being utilized by researchers to observe and quantify changes to cellular redox potential due to ROS exposure [14]. ROS can become detrimental if their concentrations increase beyond homeostatic levels due to environmental stresses, such as short-wave UV exposure, as they can react with proteins, lipids, DNA and other biomolecules, altering or inhibiting their proper functioning. It is commonly thought that the oxidative stress caused by elevated ROS levels is directly responsible for many health issues in humans, including the phenomenon of aging [15]. Therefore, the ability to monitor dynamic ROS levels in response to drugs or environmental toxicants at the microscale, and in a high throughput fashion, is of great importance. Thus development of a micropatterned cellular biosensor with the potential to detect real time shifts in ROS levels would aid in rapid screening of drugs or environmental toxicants with the capacity to modulate the redox milieu. 

In order to demonstrate a proof-of-concept cell-based biosensor, we have expanded on our previous work on cell-based bioprinting. A variety of bioprinting techniques have been extensively used by other researchers to pattern viable cells, including thermal [16,17] and piezoelectric inkjet printers [18,19,20], ablative laser printers [21,22,23,24] and extrusion-based printers [25,26]. Alternative technologies, such as printing systems designed for the patterning of microarrays, have not been fully explored for cell printing applications. These include micro-printing systems like Bioforce’s Nano eNabler^TM^ (Bioforce Nanosciences, Inc., Ames, IA, USA), as well as protein or DNA microarrayers, such as the SpotBot (Arrait Corp., Sunnyvale, CA, USA). We previously described the use of the Bioforce Nano eNabler for various bioprinting applications, including the printing of viable bacterial and mammalian cells [27]. Due to the quill-pen like nature of the printing probes used in the Nano eNabler system, we use the term “quill pen lithography” to describe this printing approach. 

One challenge for all cell-based bioprinting approaches is the effective anchoring of cells onto solid surfaces. This can require affinity-based anchoring molecules, or can be achieved by printing cells in a hydrogel-based anchoring matrix. The hydrogel matrix should be biocompatible, and ideally, tunable in composition and stiffness [28,29]. The hydrogel matrix should also remain as a liquid while still in the printer head and become a gel shortly after printing. Due to the small volumes (nanoliter and picoliter volumes) that are required, the hydrogel needs to gel quickly to prevent excessive dehydration of the droplet. One such hydrogel is based on thiolated hyaluronic acid and porcine gelatin crosslinked using polyethylene glycol diacrylate (PEGDA) (the commercial version is named HyStem^®^-C) [28]. It has been shown to allow 2D and 3D proliferation of a wide variety of stem, primary, and cancer cell types [30,31,32,33] and is composed of hyaluronic acid important for providing a life-like stem cell niche [32,34]. This formulation however gels in tens of minutes, and as such, is inappropriate for both tight spatial and temporal control of gelation. 

We report here the adaptation of both the Nano eNabler spotting apparatus and the HyStem-C hydrogel for the precise spatio-temporal spotting of a wide array of cell types and as a first step in developing live-cell-based biosensors. More specifically, we changed the manufacturing of the Nano eNabler surface patterning tool to handle larger volumes through increasing the size of its channels. In addition, the PEGDA crosslinker was replaced with PEG norbornene which allows HyStem-C to photopolymerize in seconds via radical-mediated thiol-ene step growth photopolymerization [35,36]. Finally, MHS-roGFP-R12 cells were printed, demonstrating its utility in printing a functioning, live-cell-based reactive oxygen species (ROS) sensor.

## 2. Experimental Section

### 2.1. Fabrication of the Surface Patterning Tool (SPT)

**Figure 1:**
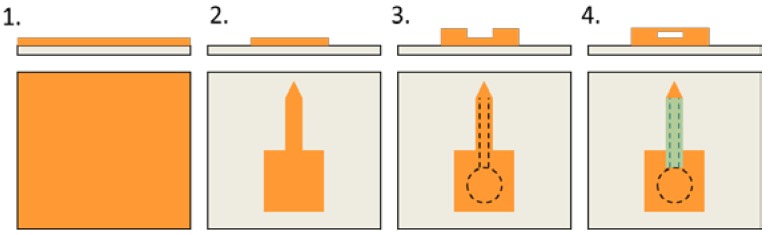
Schematic of the process flow for SU-8 SPT fabrication: (**1**) Spin on SU-8 layer; (**2**) expose device base; (**3**) spin on second SU-8 layer and expose sidewalls; (**4**) optional short exposure to form a membrane over the channel.

**Figure 2:**
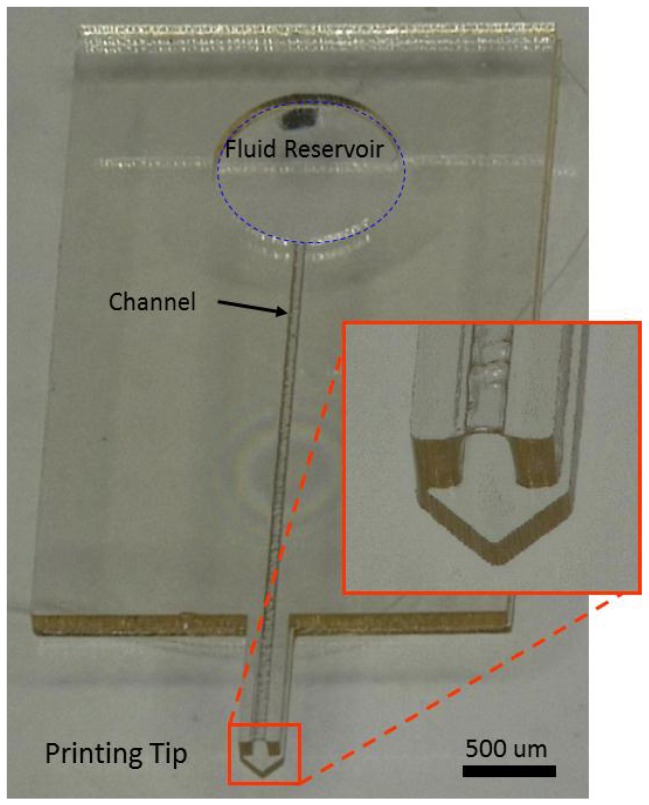
Micrograph of an SU-8 SPT with optional cover fabricated above the fluidic channel.

The printing channels of the standard “quill pen” surface patterning tools (SPTs) used with the Bioforce Nano eNabler^TM^ (NeN) are too narrow (approx. 5–40 µm in width) to accommodate the printing of mammalian cells (approximately 10 µm in diameter and larger), leading to the development of new SPTs with larger printing channels to permit cell passage. Polymeric SPTs were fabricated out of SU-8 negative photoresist using standard lithographic techniques and as described previously [27,37] and shown schematically in Figure 1. In short, the first SU-8 layer was spun onto a silicon wafer and exposed via photolithography to form the basal layer of the SPT, followed by a second resist layer that is exposed to form the reservoir and channel walls of the printing cantilever. Finally the SU-8 SPTs were released from the wafer during the SU-8 development. A micrograph of a completed SU-8 SPT is shown in Figure 2. 

### 2.2. Micro-Printer Modification

For our experiments, a Bioforce Nano eNabler^TM^ (Bioforce Nanosciences, Inc., Ames, IA, USA) was used for cell-based printing. The NeN benchtop molecular printing system was not originally optimized for live cell patterning (by the manufacturer). Some modifications to the system were required in order to maintain printed cell viability and enhance printing versatility. The NeN’s humidity sensor was removed to prevent the system from deactivating the humidifier while printing, avoiding cellular desiccation during the patterning process by maintaining >90% relative humidity within the printing enclosure. The standard SPT holder for the NeN was modified by elongating the holder’s arm to enable patterning onto the bottom of culture dishes and plates without crashing into the side walls of the dish or well. Due to this elongation, adjusting the field of view of the printing optic was required to properly image the lowered SPT during patterning. This was performed by manually lowering the objective in its housing until the proper focus at the SPT tip was achieved. When using this elongated SPT holder the tip’s angle of incidence is approximately 60° to the substrate, which allows the printing cantilever to deflect 200–400 µm after surface contact before tip failure.

### 2.3. Hydrogel Matrix Formulation and Printing Matrix Preparation

Thiolated hyaluronan (Glycosil^®^, 1% w/v), thiolated porcine gelatin (Gelin-S^®^, 1% w/v) and polyethylene glycol diacrylate (PEGDA) MW 3400 (Extralink^®^) were obtained from BioTime Inc. (Alameda, CA, USA). Irgacure^®^ 2959 was from BASF (Southfield, MI, USA). Polyethylene glycol (PEG) norbornene was derived from 4-arm PEG (MW 20,000) and was synthesized as previously described [35]. To form gels in 24-well tissue culture plates, all components (Glycosil, Gelin-S, and 1% w/v PEG norbornene) were reconstituted in 0.05% w/v Irgacure 2959 and mixed in a 2:2:1 volumetric ratio. The final concentrations (w/v) of Glycosil, Gelin-S, PEG norbornene, and Irgacure 2959 are 0.4%, 0.4%, 0.2%, and 0.05%, respectively. UV crosslinking was performed using a handheld compact UV lamp (365 nm, 4 W, 115 V, 0.16 A, Cat no. UVL-21; UVP, Upland, CA, USA). Oscillatory shear measurements of the elastic modulus, G′, and the viscous modulus, G′′, were obtained at room temperature using a Bohlin CVO rheometer (Malvern Instruments, Worcestershire, UK). Cells at the appropriate concentrations were mixed with the crosslinker in a 1:9 volumetric ratio and subsequently mixed with Glycosil and Gelin-S solutions and gelled as described above. For bioprinting, the hydrogel mixture (HyStem-C/PEGnor) was prepared as described above but with PEG-norbornene at 5% w/v initial concentration (1% w/v final concentration). The HyStem-C/PEGnor solution was then mixed with the cell-suspension at a 3:1 ratio to produce the final printing solution. 

### 2.4. Cell Culture

For initial hydrogel biocompatibility experiments, adipose derived stem cells (ADSCs) were used. ADSCs were isolated from human tumescent lipoaspiration samples as previously described [38]. Briefly, stromal vascular fraction (SVF) was prepared from human lipoaspirate. The adherent cellular fraction (adipose derived stem cells) was isolated by incubating the SVF incubated overnight at 37 °C/5% CO_2_ in control medium (Dulbecco’s modified Eagle’s Medium (DMEM), 10% fetal bovine serum (FBS), 1% antibiotic/antimycotic solution) on plastic tissue culture plates. Following incubation, the plates were washed well with 1× PBS. The remaining adherent cell population (adipose derived stem cells (ADSCs)) were cultured at 37 °C/5% CO_2_ in non-inductive control medium at sub-confluent levels.

For cell printing experiments, a variety of cell types were used, including fibroblasts, macrophages, and stem cells. NIH-3T3 murine fibroblasts were grown in DMEM medium containing 10% fetal bovine serum (FBS), 2 mM L-glutamine, and 1% penicillin streptomycin. Murine alveolar macrophages (MHS) cells were transfected with the pEGFP-N1 plasmid to allow constitutive expression of roGFP-R12 within the cytosol, while their media was supplemented with G418 antibiotic to maintain the plasmid. The resultant MHS-roGFP-R12 cells were grown in RPMI-1640 medium (Gibco^®^) supplemented with 10% FBS, 0.05 mM 2-mercaptoethanol, and 1 mg/mL G418 antibiotic. CCE mouse embryonic stem cells (mESCs) (StemCell Technologies, Vancouver, Canada) were grown on gelatin-coated tissue culture flasks in a maintenance medium consisting of Dulbecco’s Modified Eagle’s Medium (DMEM with 4.5 g/L D-glucose), supplemented with 15% (v/v) fetal bovine serum (FBS, StemCell Technology), 100 U/mL penicillin, 100 µg/mL streptomycin, 0.1 mM non-essential amino acids, 10 ng/mL murine recombinant leukemia inhibitory factor (StemCell Technology), 0.1 mM monothioglycerol, 2 mM L-glutamine, and 1 mM sodium pyruvate (Sigma-Aldrich) [39,40,41]. All cell types were incubated overnight at 37 °C/5% CO_2_. Cells were harvested at 70–80% confluency, centrifuged at 3,000×*g* for 5 min, and re-suspended in fresh growth media via vortexing to generate a suspension of single cells. 

### 2.5. Cell Printing

Qualitative confirmation of our ability to pattern viable eukaryotic cells was performed by subsequent patterning, incubation and observation of printed NIH-3T3 fibroblasts. The SU-8 SPTs selected for this experiment possessed channel widths of 100 µm and were treated with UV/ozone for 30 min to enhance the hydrophilic nature of the printing channel. After treatment, the SU-8 SPT was loaded with 2 µL of the cell/hydrogel mixture containing NIH-3T3 cells and used to pattern a glass cover-slip with a 5 × 5 array consisting of columns and rows with 500 µm spacing. After the printing process, cell-patterned cover-slips were sealed in a dish and exposed to long-wave, 365 nm UV lamp for 2 min to crosslink the HyStem-C/PEGnor hydrogel and immobilize the patterned cells. The cover-slip was then submerged in fresh media and incubated at 37 °C/5% CO_2_ until imaging was performed. Media was replaced every 24 h to maintain cell health. Bright-field imaging was performed at 18, 32, 54, 78, 100, and 194 h after patterning in order to monitor cell proliferation.

### 2.6. Examination of 3D Growth in Cell Deposits

Mouse embryonic stem cells (mESCs) were patterned and fluorescently stained in order to visualize their growth within the three dimensional (3D) microenvironment of the crosslinked droplet. The Invitrogen LIVE/DEAD^®^ Viability/Cytotoxicity kit (calcein AM and ethidium homodimer-1) for mammalian cells was used for cell staining (Carlsbad, CA, USA). These stains were selected to both visualize cells during confocal microscopy, as well as qualitatively assess the viability of the patterned mESCs. The mESCs were suspended in HyStem-C/PEGnor and printed using UV/ozone treated SU-8 SPTs onto the bottom of a glass bottom dish (WillCo Wells B.V., Amsterdam, NL, USA) in a 5 × 5 array (500 µm spacings). The patterned dish was then sealed and exposed to UV light for 2 min, to crosslink the HyStem-C/PEGnor hydrogel and immobilize the patterned cells. The cell array was then submerged in fresh media and incubated for 72 h at 37 °C/5% CO_2_, with media being refreshed every 24 h. At hour 72 confocal laser scanning microscopy (Leica SP-5, Leica Microsystems) was performed to gather three-dimensional information on the cell growth occurring within the printed spots.

### 2.7. Development of a Living ROS Sensor

MHS-roGFP-R12 was developed essentially as described by Melillo *et al*. [42]. MHS-roGFP-R12 cell-laden hydrogel mixture was loaded into UV/ozone treated SU-8 SPTs and deposited in a 5 × 5 array (500 µm spacings) onto the bottom of a glass bottom dish (WillCo Wells B.V.). The patterned dish was then sealed and UV-crosslinked for 2 min to immobilize cell deposits. After exposure, the dish was opened and the cell array was rinsed three times with 1× HBSS buffer (Gibco^®^), after which fresh media was added to the dish, submerging the printed array. The added media lacked any FBS to prevent neutralization of ROS in solution during the detection trials. Following addition of media, the dish was mounted on a confocal laser scanning microscope (Leica SP5, Leica Microsystems) and the cell array was imaged by fixing the emission wavelength at 515 nm while the excitation wavelength was toggled between 405 and 488 nm, to quantify oxidization and reduction fluorescence shifts, respectively. After base-line images were gathered at 1 min intervals for 5 min, the cells were exposed to 500 µM of hydrogen peroxide and imaged at 1 min intervals for a period of 8 min. Cells were then exposed to 10 mM of Dithiothreitol (DTT) in order to neutralize oxidizing activity and return cellular roGFP to a fully reduced state, followed by imaging at 1 min intervals for 4 min. Results gathered from individual cells were quantified using Leica LAS image processing software and plotted to determine if shifts in roGFP excitation would directly correspond to the addition of oxidizers and reducers to the environment.

## 3. Results and Discussion

### 3.1. Hydrogel Matrix Formulation and Optimization

**Figure 3:**
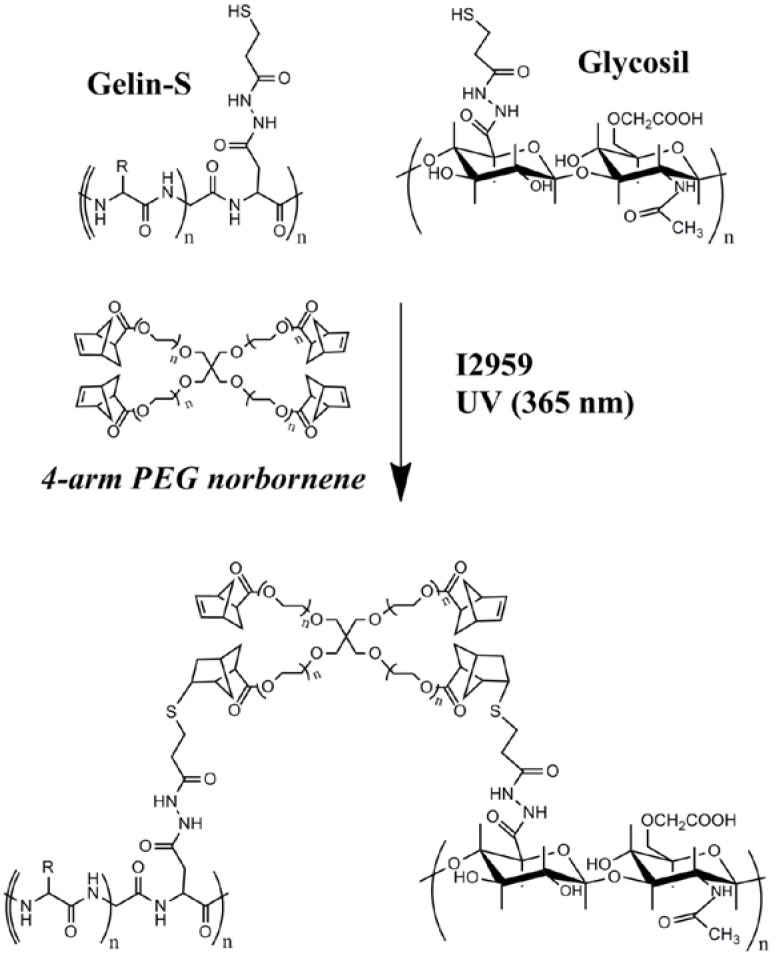
Radically-mediated thiol-ene photopolymerization using thiolated hyaluronic acid, porcine gelatin, 4-arm PEG norbornene, and Irgacure 2959 (I2959).

Initial bioprinting experiments with HyStem^®^-C showed that we did not have the requisite temporal control of gelation for bioprinting on glass cover-slips using the modified SPT in the Nano eNabler device. More specifically, HyStem-C gelled too slowly after spotting. This constraint required close attention to maintenance of chamber humidity; in addition, spotting experiments were slowed since we were required to wait for gelation before adding media. Another limitation was the hydrogel’s spontaneous crosslinking. Since crosslinking typically occurs via the Michael Addition reaction between the hydrogel thiols and the PEGDA acrylates, the reaction gels in 10–20 min and can potentially clog the SPT channels mid-experiment. The solution was to substitute the polyethylene glycol (PEG)-diacrylate crosslinker with PEG-norbornene [35], which can be photo-coupled to thiol-containing molecules using radical-initiated “thiol-ene” chemistry [43]. Specifically, radically initiated thiyl radicals form from the cysteines of the thiolated hyaluronic acid (Glycosil) and porcine gelatin (Gelin-S) components in the presence of photoinitiator (Irgacure 2959) and ultraviolet light. The thiyl groups couple through the alkene bond of the norbornene adducts on the ends of four-armed PEG molecule (Figure 3). 

**Figure 4:**
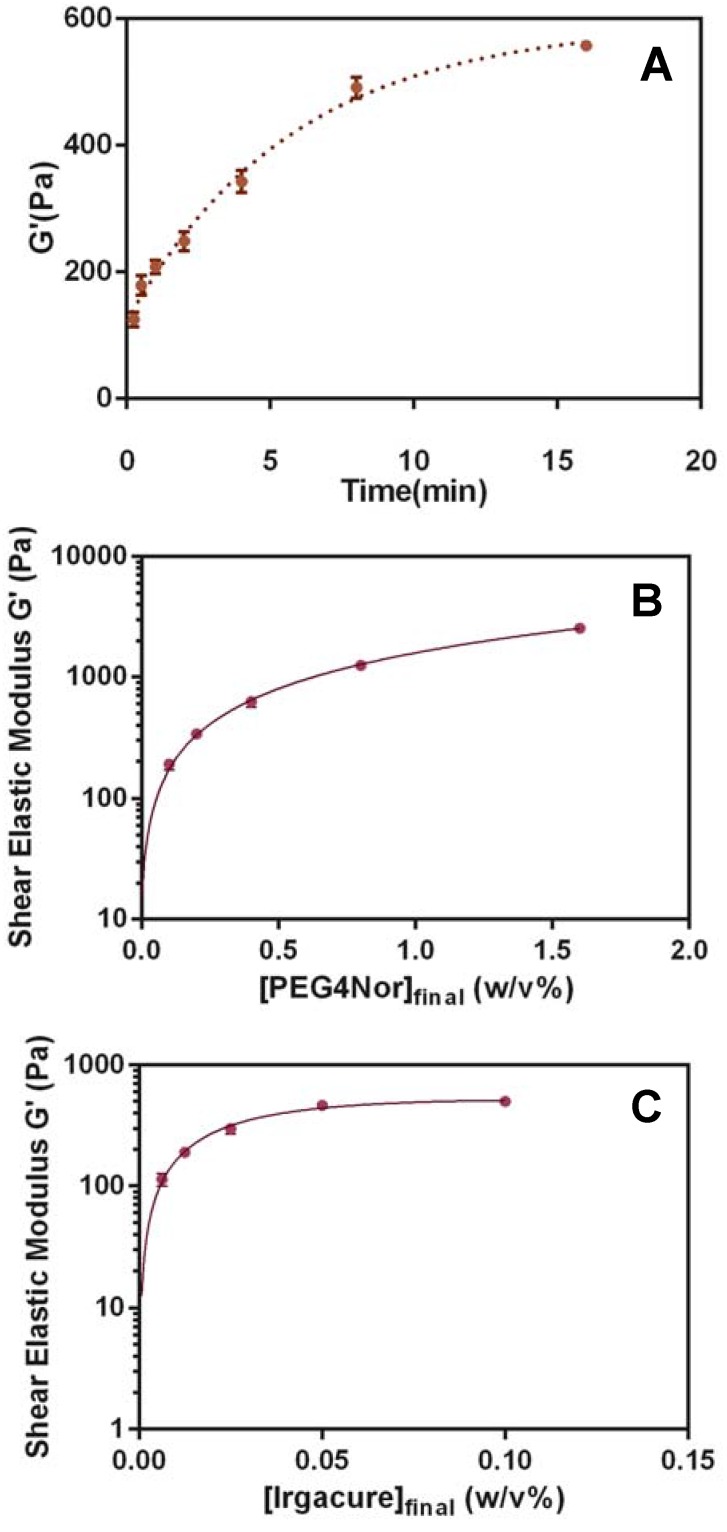
Rheometry experiments for shear elastic modulus (G′) of hydrogel networks formed with Glycosil, Gelin-S, at different ultraviolet light exposure times and substrate concentrations. (**A**) Time course with final concentration 0.2% w/v PEG norbornene and UV illumination for up to 15 min. (**B**) PEG norbornene dose response (final concentrations listed) with UV illumination for 3 minutes at each concentration. (**C**) Irgacure 2959 dose response with hydrogels prepared with PEG norbornene (0.2% final concentration) and UV illuminated for 3 min at each concentration.

Using thiol-ene chemistry, hydrogels were formed within thirty seconds and attained a shear elastic modulus (G′) of up to 550 Pa after 15 min with only a final concentration of 0.2% w/v PEG norbornene (Figure 4(A)). The gelation occurred at every ratio of thiols and alkenes (from thiol:alkene molar ratios of 31 down to 1.94 depending on the PEG norbornene concentration used (Figure 4(B)). Importantly, gel stiffness could also be modulated with increasing concentration of PEG norbornene to a shear elastic modulus greater than 2 kPa (Figure 4(C)). The optimized Irgacure 2959 concentration is 0.05% (Figure 4(C)). Therefore, photoinitiated thiol-ene chemistry enabled rapid gelation of thiolated hyaluronan/gelatin hydrogels, thus providing a matrix that is well-suited for bioprinting.

### 3.2. Biocompatibility of the HyStem-C/PEG Norbornene Hydrogels

**Figure 5:**
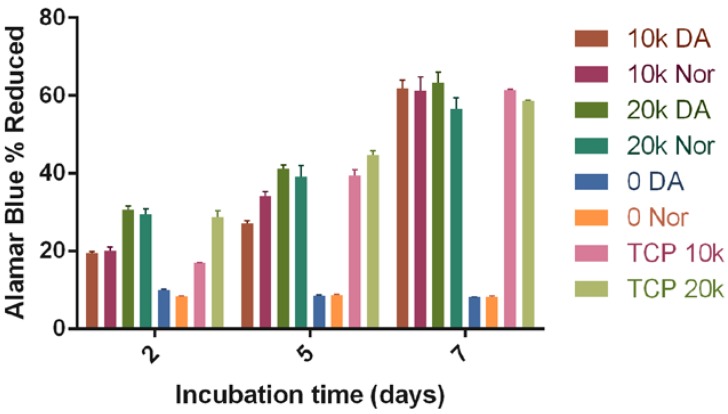
Alamar Blue percent reduction for ADSCs on HyStem-C crosslinked with either PEGDA (Extralink) or PEG norbornene and grown in 2D. The figure legend nomenclature shows number of cells/well (0; 10 K, 10,000; 20 K, 20,000) followed by type of crosslinker (DA, Extralink; Nor, PEG norbornene). TCP indicates tissue culture plastic controls at different cell numbers.

Biocompatibility of the HyStem-C/PEG norbornene hydrogels was explored using human adipose-derived stem cells (ADSCs) isolated from human lipoaspirates. Human ADSCs were chosen for these experiments due to their clinical potential (e.g., for tissue engineering and cell therapy [44,45]). ADSCs were cultured on top of either HyStem-C/PEG norbornene or HyStem-C hydrogels (the latter as a control). The proliferation rates between cells at two different cell numbers was similar for both hydrogels after one week (Figure 5), indicating that neither the UV exposure nor presence of PEG norbornene and Irgacure 2959 presented cytotoxicity issues at the UV intensities used.

### 3.3. Cell Proliferation after Printing

**Figure 6:**
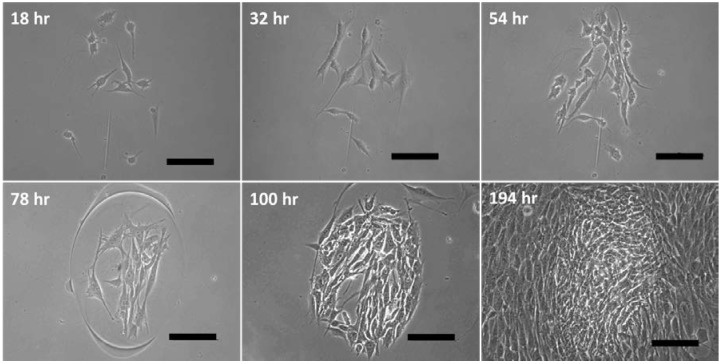
Demonstration of NIH/3T3 cell growth within printed spots over 8 days, images at hour 18, 32, and 54 show the consistent progression of the same spot. Scale bar = 100 μm.

For cell printing experiments, we first attempted printing of a robust fibroblast line, namely NIH-3T3 cells. NIH-3T3 cells were printed in the HyStem-C/PEG norbornene hydrogel and imaged at several time points during their growth, starting at 18 h and ending at 192 h, when the cells began to overgrow the surface (Figure 6). Directly after patterning, cells possessed a rounded morphology, similar to that displayed by unattached cells suspended in solution. However, images at 18 h show cells beginning to spread and form long pseudopodia, indicative of cell attachment to either the glass surface or the 3D HyStem-C/PEGnor matrix (Figure 6). As growth progressed through 32 h, cells began proliferating, and at 100 h started to escape from the printed matrix and spread across the glass substrate. By 192 h the cells had completely over-grown the entire array area (4 mm^2^), obscuring the array and discouraging further imaging. At 192 h the original depositions remained visible, due to the higher density of cells within the 3D matrix, as compared to the confluent cells growing on the interstitial, two dimensional glass surface.

### 3.4. The 3D Nature of the Printed Cell Matrix

**Figure 7:**
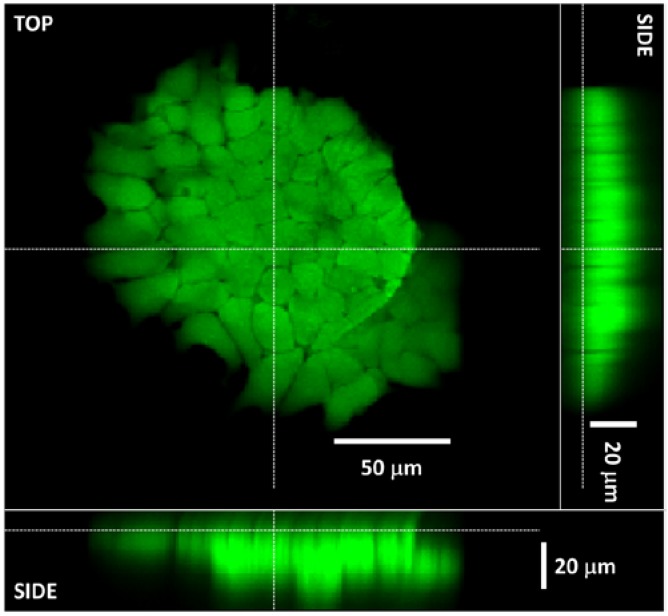
Demonstration of viable mESC patterning into discrete, 3D spots via live/dead staining and confocal laser scanning microscopy. Image cross-section suggests cells are distributed in the Z-direction, in addition to the XY-plane.

To assess the 3D nature of printed spots of cells in HyStem-C/PEG norbornene, and further assess biocompatibility with other cell lines, we printed mouse embryonic stem cells (mESCs). Patterned mESCs were subjected to live/dead staining. After staining, confocal laser scanning microscopy was used to reveal the cell positions within the printed matrix material by producing an optical cross-section of the deposition from the complied image planes (Figure 7). Examination of the deposition cross-section confirms that the cells growing within the matrix reach a height of approximately 20–25 µm from the glass surface. Like many eukaryotic cells, the average mESC cell possesses a diameter of roughly 10 µm, suggesting that the growth within the microenvironment of the printed matrix is two to three cell layers in height, which is indicative of 3D cell growth. Additionally, none of the cells within the examined deposition were positive for ethidium homodimer-1 (which stains the nucleus red for dead cells), demonstrating that high survivability of mESCs was maintained when submitted to our patterning process.

### 3.5. Verification of Living ROS Sensor Function

**Figure 8:**
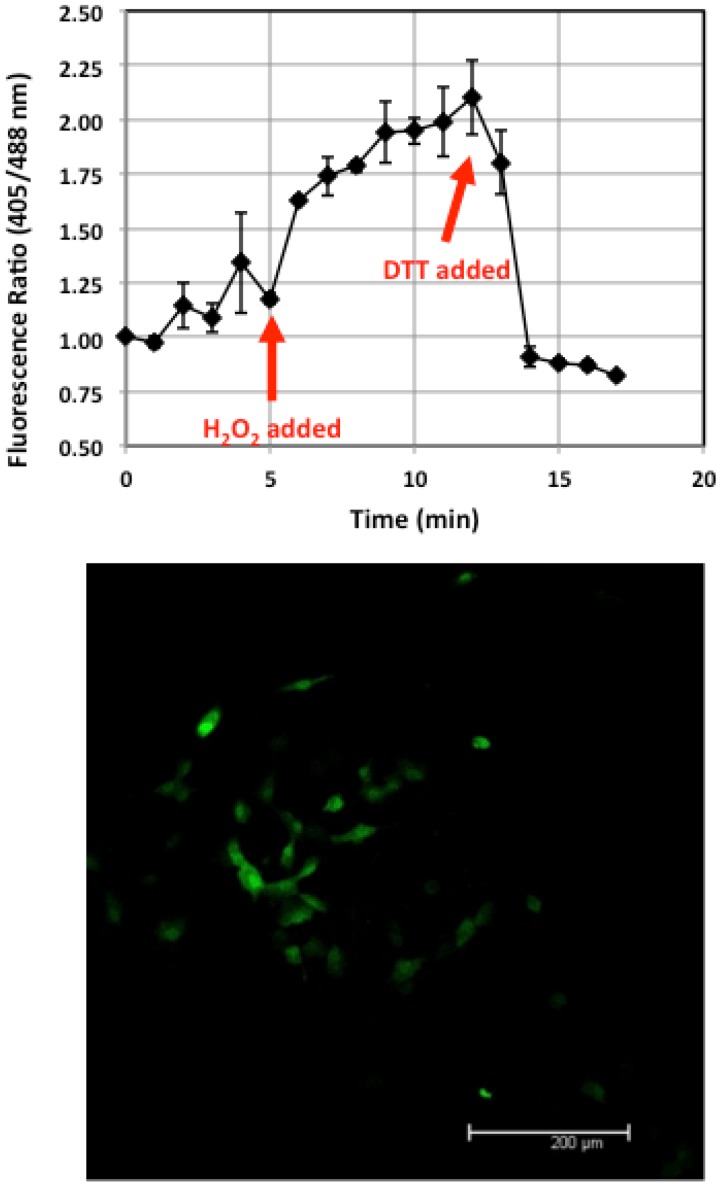
**Top**: Living biosensor function is verified by plotting the ratio of 405 nm fluorescence intensity (oxidized) over the 488 nm intensity (reduced). **Bottom**: Fluorescence micrograph of a representative printed spot of MHS-roGFP-R12 cells (scale 200 µm).

The ROS sensing capability of the printed MHS-roGFP-R12 cells was tested by exposing the cell array to oxidizing agent (hydrogen peroxide), followed by exposure to reducing agent (DTT). Cell response was quantified by selecting three separate cells within a single printed spot for observation. Utilizing confocal scanning laser microscopy in conjunction with Leica LAS image processing software, the resulting intensity at 515 nm was measured for each cell at 1 min intervals during exposure. The MHS-roGFP-R12 cells demonstrate maximum fluorescence emission at 515 nm when excited by 405 nm, if the roGFP-R12 protein is in the oxidized form. Likewise, they demonstrate maximum excitation at 488 nm when in the reduced state. Thus, by exciting cells at both 405 and 488 nm wavelengths and then taking the ratio of resulting emission at 515 nm (for both excitation wavelengths), one can deduce the relative state (oxidized *vs*. reduced) for the roGFP-R12 protein [14]. Ratiometric comparison of oxidized to reduced roGFP-R12 was performed for each of three cells, individually, which were then averaged together in order to quantify the overall response from the spot as a whole (Figure 8). Examining these data reveals that the printed cells were able to respond to both oxidizing and reducing environments rapidly and effectively. Results from all three cells demonstrate an oxidative response directly upon addition of hydrogen peroxide (minute 5), which increases toward a maximal level until a reductive response is elicited immediately upon addition of reductive DTT (minute 13). This result confirms the proper functioning of the patterned MHS-roGFP-R12 cells, as well as the capability to print a functioning and reversible live-cell-based ROS sensor.

## 4. Discussion

Current bioprinting technologies are most commonly used to engineer and fabricate cell-based constructs for tissue engineering applications [1]. Some techniques, such as inkjet bioprinting, have been used to develop high-throughput drug screening platforms [4], however comparatively little has been done toward the development of cell-based biosensors using bioprinting. A potential barrier to bioprinting for cell-based biosensor fabrication is the acquisition of specialized equipment and the experience required in order to use said equipment to reliably pattern viable cells. We have successfully modified a bench-top micro-patterning system, and in turn developed a novel method for printing live eukaryotic cells, a method we have entitled quill pen lithography (QPL). In addition, we have modified a biocompatible hyaluronic acid based hydrogel, HyStem-C, to include a new crosslinker (PEGnor) which provides the requisite spatial-temporal control for bioprinting applications. The resulting HyStem-C/PEG norbornene hydrogels were shown to be biocompatible with human derived adipose-derived stem cells, which demonstrate the possible utility for future tissue engineering applications. We also showed that the elastic modulus of these hydrogels was dynamically tunable across a range of 550 Pa to >2 kPa, which is within a range of several human tissues [46]. 

Cell printing experiments demonstrated that mammalian cells (NIH-3T3) could both survive the printing process and proliferate during long-term culture. Cells remained within the printed spots until later time points (>100 h) but eventually escaped the 3D hydrogel matrix. Importantly, our experiments with mESCs printed in HyStem-C/PEG norbornene survived printing (as determined by live/dead staining) and grew both vertically and laterally within the matrix, as confocal microscopy showed cells growing throughout the height of the spot (>20 µm). Our results demonstrate that HyStem-C/PEG norbornene matrices are suitable for 3D cellular organization, and the utility of our cell printing method for producing small, 3D cell cultures. 

Using our printing approach, we have also developed a functional, living ROS biosensor by immobilizing cells that express a redox sensitive, fluorescent protein to a surface which permits easy observation and quantification of the intensity shifts which occur upon changes in environmental redox levels. The success of our 3D bioprinting approach for cell-based biosensing indicates that the QPL technique is a viable alternative to other bioprinting strategies for the development of 2D platforms for studying both cell sensing and signaling phenomena. While this sensor was a proof of concept device, the same fabrication technique can used to develop similar, more sophisticated devices with the capability of monitoring intracellular redox activity. By incorporating cells that have been engineered to express roGFP at a specific location, (e.g., in the mitochondria or cell membrane) redox events which occur at the organelle can be monitored and quantified, potentially elucidating ROS signaling mechanisms or their cytotoxic effects. In addition to observing the redox activity of ROS in a controlled environment, this living sensor could be used conversely to perform high-throughput screening of a variety of antioxidants for counteracting the presence or activity of these ROS molecules *in vitro*. More advanced applications of such a sensor could also include evaluating the proximity effects of ROS production from natural sources, such as senescent cells [47] on the physiology of nearby, healthy cells.

Finally, our results demonstrate that micro-printing tools already useful for the fabrication of protein and DNA-based biosensors, in this case the NeN, can be repurposed as viable cell printers for the development of cell-based sensors. This would be especially useful for groups who utilize these tools on a regular basis as the required modifications can be performed rapidly (in minutes) and without specialized tools or knowledge. The only additional components needed to transform the NeN into an effective cell printer are the SU-8 SPTs described here that possess the necessary dimensions to transfer whole cells onto solid and semi-solid surfaces.

## 5. Conclusions

We have developed a novel, direct cell-patterning technique using a modified protein microarray printing system in conjunction with a specialized surface patterning tool and hydrogel specifically formulated to immobilize cells in a highly biocompatible and cytocompatible 3D matrix. Viable cell printing using QPL was verified by monitoring the resulting cell growth and morphology after patterning via microscopy, as well through qualitative viability staining. The 3D nature of the crosslinked cell depositions was revealed via optical cross-sectioning using confocal laser scanning microscopy, demonstrating that each spot is actually a 3D microenvironment in which the cells are not restricted to motility solely in the XY-plane, but are also able to move in the Z-plane. The utility of QPL bioprinting was demonstrated by fabricating a live-cell-based biosensor that is capable of monitoring changes in redox activity within an engineered *in vitro* environment.
